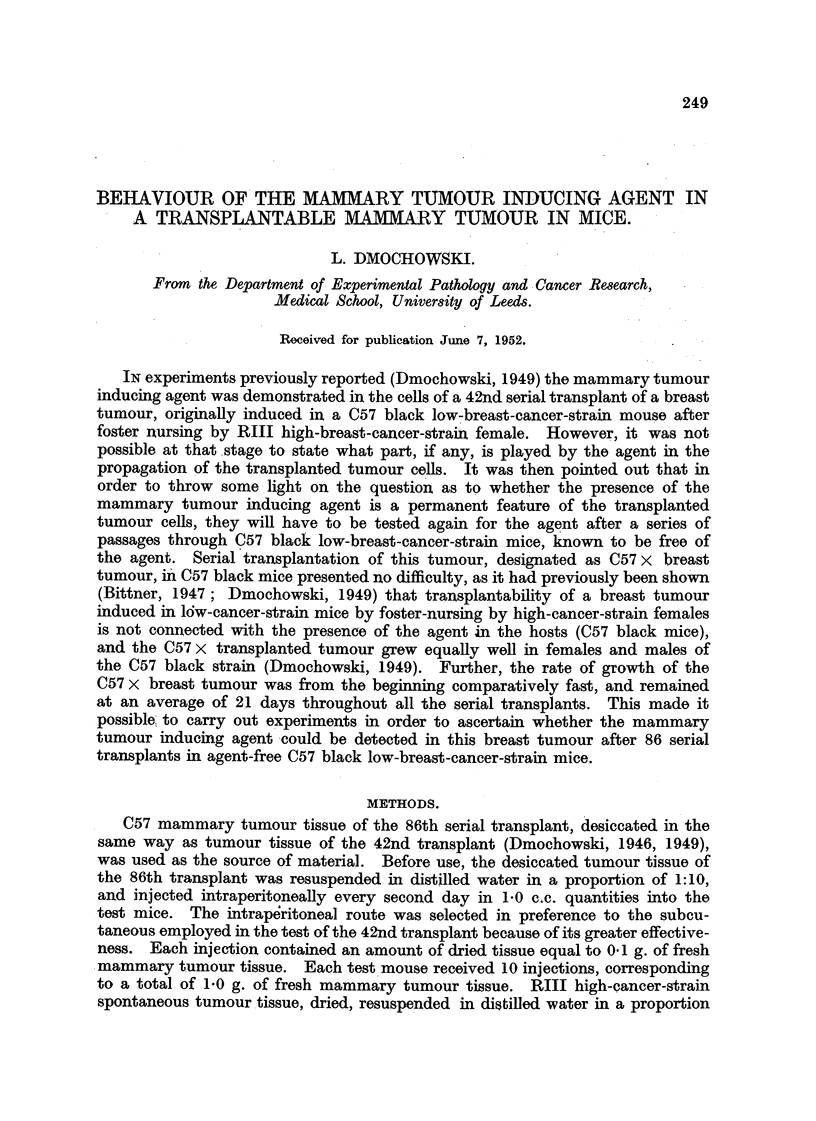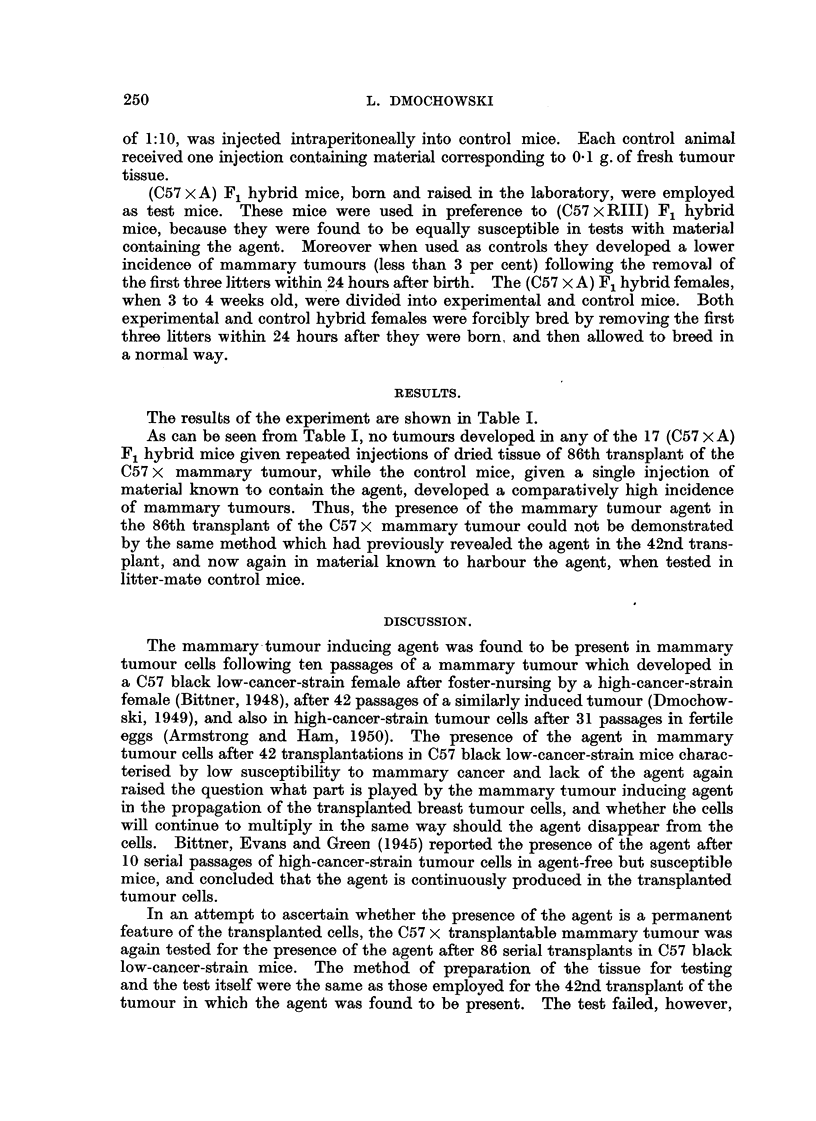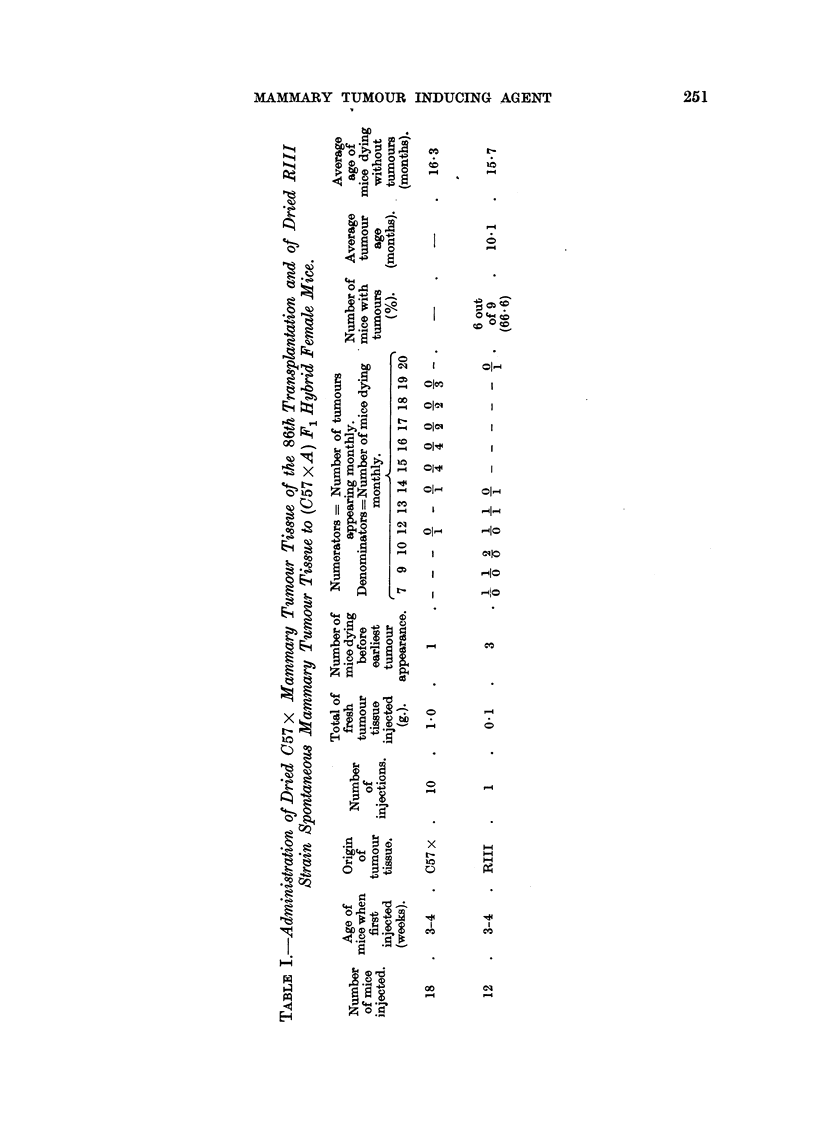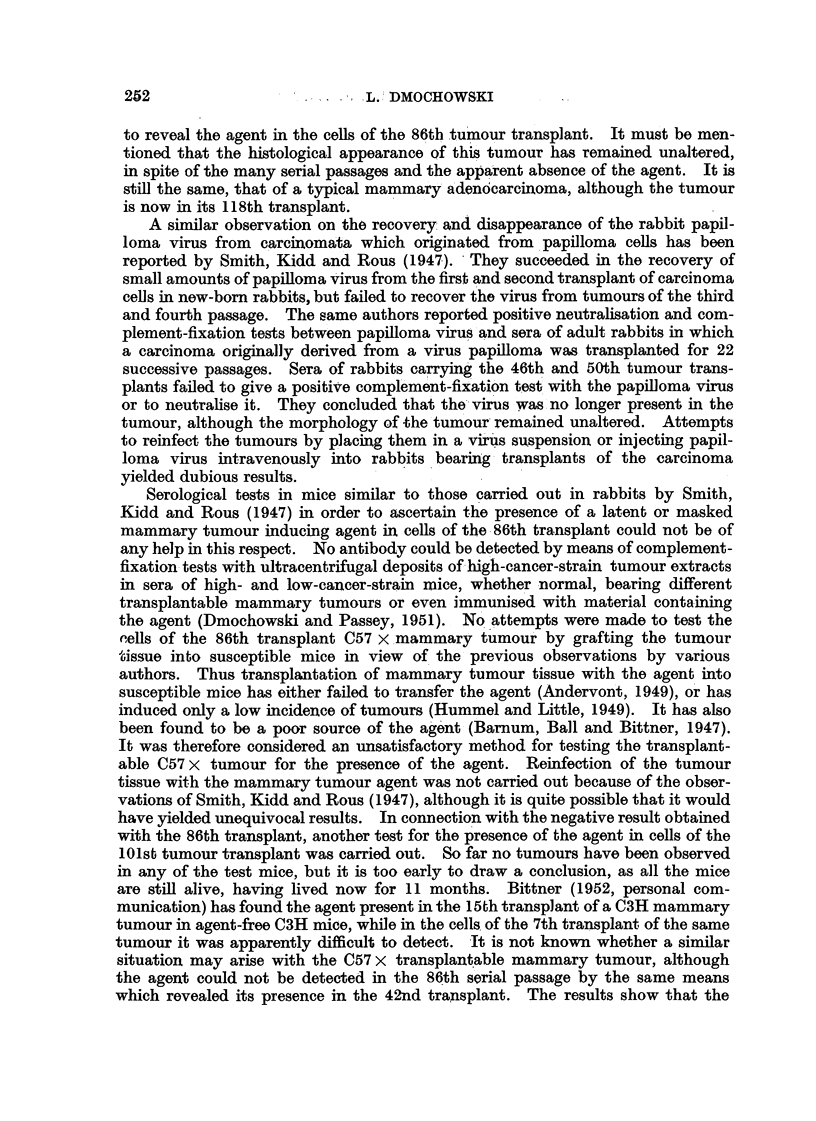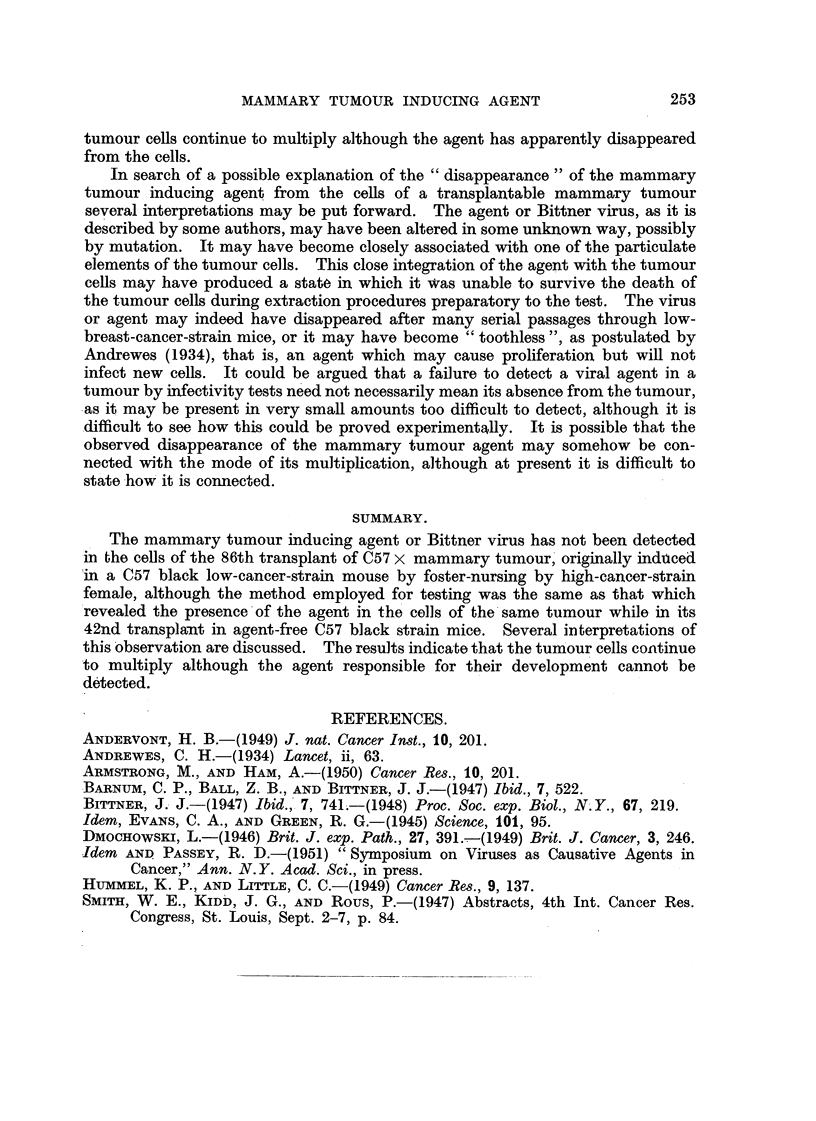# Behaviour of the Mammary Tumour Inducing Agent in a Transplantable Mammary Tumour in Mice

**DOI:** 10.1038/bjc.1952.29

**Published:** 1952-09

**Authors:** L. Dmochowski


### BEHAVIOUR OF THE MAMMARY TUMOUR INDUCING AGENT IN A TRANSPLANTABLE MAMMARY TUMOUR IN MICE.

L. DMOCHOWSKI.

*From the Department of Experimental Pathology and Cancer Research, Medical School, University of Leeds.*

Received for publication June 7, 1952.

IN experiments previously reported (Dmochowski, 1949) the mammary tumour inducing agent was demonstrated in the cells of a 42nd serial transplant of a breast tumour, originally induced in a C57 black low-breast-cancer-strain mouse after foster nursing by RIII high-breast-cancer-strain female. However, it was not possible at that stage to state what part, if any, is played by the agent in the propagation of the transplanted tumour cells. It was then pointed out that in order to throw some light on the question as to whether the presence of the mammary tumour inducing agent is a permanent feature of the transplanted tumour cells, they will have to be tested again for the agent after a series of passages through C57 black low-breast-cancer-strain mice, known to be free of the agent. Serial transplantation of this tumour, designated as C57 × breast tumour, in C57 black mice presented no difficulty, as it had previously been shown (Bittner, 1947 ; Dmochowski, 1949) that transplantability of a breast tumour induced in low-cancer-strain mice by foster-nursing by high-cancer-strain females is not connected with the presence of the agent in the hosts (C57 black mice), and the C57 × transplanted tumour grew equally well in females and males of the C57 black strain (Dmochowski, 1949). Further, the rate of growth of the C57 × breast tumour was from the beginning comparatively fast, and remained at an average of 21 days throughout all the serial transplants. This made it possible to carry out experiments in order to ascertain whether the mammary tumour inducing agent could be detected in this breast tumour after 86 serial transplants in agent-free C57 black low-breast-cancer-strain mice.

### METHODS.

C57 mammary tumour tissue of the 86th serial transplant, desiccated in the same way as tumour tissue of the 42nd transplant (Dmochowski, 1946, 1949), was used as the source of material. Before use, the desiccated tumour tissue of the 86th transplant was resuspended in distilled water in a proportion of 1:10, and injected intraperitoneally every second day in 1·0 c.c. quantities into the test mice. The intraperitoneal route was selected in preference to the subcutaneous employed in the test of the 42nd transplant because of its greater effectiveness. Each injection contained an amount of dried tissue equal to 0·1 g. of fresh mammary tumour tissue. Each test mouse received 10 injections, corresponding to a total of 1·0 g. of fresh mammary tumour tissue. RIII high-cancer-strain spontaneous tumour tissue, dried, resuspended in distilled water in a proportion of 1:10, was injected intraperitoneally into control mice. Each control animal received one injection containing material corresponding to 0·1 g. of fresh tumour tissue.

(C57 × A) $F_1$ hybrid mice, born and raised in the laboratory, were employed as test mice. These mice were used in preference to (C57 × RIII) $F_1$ hybrid mice, because they were found to be equally susceptible in tests with material containing the agent. Moreover when used as controls they developed a lower incidence of mammary tumours (less than 3 per cent) following the removal of the first three litters within 24 hours after birth. The (C57 × A) $F_1$ hybrid females, when 3 to 4 weeks old, were divided into experimental and control mice. Both experimental and control hybrid females were forcibly bred by removing the first three litters within 24 hours after they were born, and then allowed to breed in a normal way.

### RESULTS.

The results of the experiment are shown in Table I.

As can be seen from Table I, no tumours developed in any of the 17 (C57 × A) $F_1$ hybrid mice given repeated injections of dried tissue of 86th transplant of the C57 × mammary tumour, while the control mice, given a single injection of material known to contain the agent, developed a comparatively high incidence of mammary tumours. Thus, the presence of the mammary tumour agent in the 86th transplant of the C57 × mammary tumour could not be demonstrated by the same method which had previously revealed the agent in the 42nd transplant, and now again in material known to harbour the agent, when tested in litter-mate control mice.

### DISCUSSION.

The mammary tumour inducing agent was found to be present in mammary tumour cells following ten passages of a mammary tumour which developed in a C57 black low-cancer-strain female after foster-nursing by a high-cancer-strain female (Bittner, 1948), after 42 passages of a similarly induced tumour (Dmochowski, 1949), and also in high-cancer-strain tumour cells after 31 passages in fertile eggs (Armstrong and Ham, 1950). The presence of the agent in mammary tumour cells after 42 transplantations in C57 black low-cancer-strain mice characterised by low susceptibility to mammary cancer and lack of the agent again raised the question what part is played by the mammary tumour inducing agent in the propagation of the transplanted breast tumour cells, and whether the cells will continue to multiply in the same way should the agent disappear from the cells. Bittner, Evans and Green (1945) reported the presence of the agent after 10 serial passages of high-cancer-strain tumour cells in agent-free but susceptible mice, and concluded that the agent is continuously produced in the transplanted tumour cells.

In an attempt to ascertain whether the presence of the agent is a permanent feature of the transplanted cells, the C57 × transplantable mammary tumour was again tested for the presence of the agent after 86 serial transplants in C57 black low-cancer-strain mice. The method of preparation of the tissue for testing and the test itself were the same as those employed for the 42nd transplant of the tumour in which the agent was found to be present. The test failed, however,

TABLE I.—*Administration of Dried C57 × Mammary Tumour Tissue of the 86th Transplantation and of Dried RIII Strain Spontaneous Mammary Tumour Tissue to (C57 × A) $F_1$ Hybrid Female Mice.*

| Number of mice injected. | Age of mice when first injected (weeks). | Origin of tumour tissue. | Number of injections. | Total of fresh tumour tissue injected (g.). | Number of mice dying before earliest tumour appearance. | Numerators = Number of tumours appearing monthly. Denominators = Number of mice dying monthly. | | | | | | | | | | | | Number of mice with tumours (%). | Average tumour age (months). | Average age of mice dying without tumours (months). |
|---|---|---|---|---|---|---|---|---|---|---|---|---|---|---|---|---|---|---|---|---|
| | | | | | | 7 | 9 | 10 | 12 | 13 | 14 | 15 | 16 | 17 | 18 | 19 | 20 | | | |
| 18 | 3–4 | C57 × | 10 | 1·0 | 1 | – | – | – | $\frac{0}{1}$ | – | $\frac{0}{1}$ | $\frac{0}{4}$ | $\frac{0}{4}$ | $\frac{0}{2}$ | $\frac{0}{2}$ | $\frac{0}{3}$ | – | — | — | 16·3 |
| 12 | 3–4 | RIII | 1 | 0·1 | 3 | $\frac{1}{0}$ | $\frac{1}{0}$ | $\frac{2}{0}$ | $\frac{1}{0}$ | $\frac{1}{1}$ | $\frac{0}{1}$ | – | – | – | – | – | $\frac{0}{1}$ | 6 out of 9 (66·6) | 10·1 | 15·7 |

to reveal the agent in the cells of the 86th tumour transplant. It must be mentioned that the histological appearance of this tumour has remained unaltered, in spite of the many serial passages and the apparent absence of the agent. It is still the same, that of a typical mammary adenocarcinoma, although the tumour is now in its 118th transplant.

A similar observation on the recovery and disappearance of the rabbit papilloma virus from carcinomata which originated from papilloma cells has been reported by Smith, Kidd and Rous (1947). They succeeded in the recovery of small amounts of papilloma virus from the first and second transplant of carcinoma cells in new-born rabbits, but failed to recover the virus from tumours of the third and fourth passage. The same authors reported positive neutralisation and complement-fixation tests between papilloma virus and sera of adult rabbits in which a carcinoma originally derived from a virus papilloma was transplanted for 22 successive passages. Sera of rabbits carrying the 46th and 50th tumour transplants failed to give a positive complement-fixation test with the papilloma virus or to neutralise it. They concluded that the virus was no longer present in the tumour, although the morphology of the tumour remained unaltered. Attempts to reinfect the tumours by placing them in a virus suspension or injecting papilloma virus intravenously into rabbits bearing transplants of the carcinoma yielded dubious results.

Serological tests in mice similar to those carried out in rabbits by Smith, Kidd and Rous (1947) in order to ascertain the presence of a latent or masked mammary tumour inducing agent in cells of the 86th transplant could not be of any help in this respect. No antibody could be detected by means of complement-fixation tests with ultracentrifugal deposits of high-cancer-strain tumour extracts in sera of high- and low-cancer-strain mice, whether normal, bearing different transplantable mammary tumours or even immunised with material containing the agent (Dmochowski and Passey, 1951). No attempts were made to test the cells of the 86th transplant C57 × mammary tumour by grafting the tumour tissue into susceptible mice in view of the previous observations by various authors. Thus transplantation of mammary tumour tissue with the agent into susceptible mice has either failed to transfer the agent (Andervont, 1949), or has induced only a low incidence of tumours (Hummel and Little, 1949). It has also been found to be a poor source of the agent (Barnum, Ball and Bittner, 1947). It was therefore considered an unsatisfactory method for testing the transplantable C57 × tumour for the presence of the agent. Reinfection of the tumour tissue with the mammary tumour agent was not carried out because of the observations of Smith, Kidd and Rous (1947), although it is quite possible that it would have yielded unequivocal results. In connection with the negative result obtained with the 86th transplant, another test for the presence of the agent in cells of the 101st tumour transplant was carried out. So far no tumours have been observed in any of the test mice, but it is too early to draw a conclusion, as all the mice are still alive, having lived now for 11 months. Bittner (1952, personal communication) has found the agent present in the 15th transplant of a C3H mammary tumour in agent-free C3H mice, while in the cells of the 7th transplant of the same tumour it was apparently difficult to detect. It is not known whether a similar situation may arise with the C57 × transplantable mammary tumour, although the agent could not be detected in the 86th serial passage by the same means which revealed its presence in the 42nd transplant. The results show that the tumour cells continue to multiply although the agent has apparently disappeared from the cells.

In search of a possible explanation of the " disappearance " of the mammary tumour inducing agent from the cells of a transplantable mammary tumour several interpretations may be put forward. The agent or Bittner virus, as it is described by some authors, may have been altered in some unknown way, possibly by mutation. It may have become closely associated with one of the particulate elements of the tumour cells. This close integration of the agent with the tumour cells may have produced a state in which it was unable to survive the death of the tumour cells during extraction procedures preparatory to the test. The virus or agent may indeed have disappeared after many serial passages through low-breast-cancer-strain mice, or it may have become " toothless ", as postulated by Andrewes (1934), that is, an agent which may cause proliferation but will not infect new cells. It could be argued that a failure to detect a viral agent in a tumour by infectivity tests need not necessarily mean its absence from the tumour, as it may be present in very small amounts too difficult to detect, although it is difficult to see how this could be proved experimentally. It is possible that the observed disappearance of the mammary tumour agent may somehow be connected with the mode of its multiplication, although at present it is difficult to state how it is connected.

SUMMARY.

The mammary tumour inducing agent or Bittner virus has not been detected in the cells of the 86th transplant of C57 × mammary tumour, originally induced in a C57 black low-cancer-strain mouse by foster-nursing by high-cancer-strain female, although the method employed for testing was the same as that which revealed the presence of the agent in the cells of the same tumour while in its 42nd transplant in agent-free C57 black strain mice. Several interpretations of this observation are discussed. The results indicate that the tumour cells continue to multiply although the agent responsible for their development cannot be detected.

### REFERENCES.

ANDERVONT, H. B.—(1949) *J. nat. Cancer Inst.*, **10**, 201.
ANDREWES, C. H.—(1934) *Lancet*, ii, 63.
ARMSTRONG, M., AND HAM, A.—(1950) *Cancer Res.*, **10**, 201.
BARNUM, C. P., BALL, Z. B., AND BITTNER, J. J.—(1947) *Ibid.*, **7**, 522.
BITTNER, J. J.—(1947) *Ibid.*, **7**, 741.—(1948) *Proc. Soc. exp. Biol., N.Y.*, **67**, 219.
*Idem*, EVANS, C. A., AND GREEN, R. G.—(1945) *Science*, **101**, 95.
DMOCHOWSKI, L.—(1946) *Brit. J. exp. Path.*, **27**, 391.—(1949) *Brit. J. Cancer*, **3**, 246.
*Idem* AND PASSEY, R. D.—(1951) " Symposium on Viruses as Causative Agents in Cancer," *Ann. N.Y. Acad. Sci.*, in press.
HUMMEL, K. P., AND LITTLE, C. C.—(1949) *Cancer Res.*, **9**, 137.
SMITH, W. E., KIDD, J. G., AND ROUS, P.—(1947) Abstracts, 4th Int. Cancer Res. Congress, St. Louis, Sept. 2–7, p. 84.